# Apparent Clitoromegaly in a Newborn: A Case of Congenital Adrenal Hyperplasia

**DOI:** 10.7759/cureus.89311

**Published:** 2025-08-03

**Authors:** Mariam Svanadze, Tinatin Gagua, Tamar Svanadze, Alexandra Barnovi, Mariam Morchadze

**Affiliations:** 1 Obstetrics and Gynaecology, Gagua Clinic, Tbilisi, GEO; 2 Obstetrics and Gynaecology, David Tvildiani Medical University, Gagua Clinic, Tbilisi, GEO; 3 Pediatrics, University of Georgia, Tbilisi, GEO; 4 Gynecologic Oncology, University of Georgia, Tbilisi, GEO

**Keywords:** clitoromegaly, congenital adrenal hyperplasia, congenital anomaly, disorder of sex development (dsd), endocrinology, gynecology, newborn, paediatrics

## Abstract

This case report elucidates the diagnostic trajectory of a female newborn, presenting with apparent clitoromegaly, ultimately diagnosed with congenital adrenal hyperplasia (CAH). The patient was born in a prominent obstetrics and gynecology center in Tbilisi, Georgia, where the anomaly was promptly identified following a physiologically normal pregnancy and labor. Despite the relative infrequency of such cases in our center, particularly among term infants, the handling of this case was swift and successful. Comprehensive examinations were conducted, with meticulous documentation of clitoromegaly measurements utilizing contemporary methodologies and grading scales. The center conducted essential primary tests, including abdominal and pelvic ultrasounds, and advised the parents on the requisite laboratory tests to ascertain a precise diagnosis. Laboratory tests mainly focused on detecting any abnormality in the pituitary-adrenal gland axis functioning. However, the delay in obtaining karyotyping due to its high cost did not hinder the diagnostic process. Pertinent findings collectively led to the diagnosis of 46 (XX) disorder of sex development (DSD), specifically CAH. Subsequently, the patient's parents received comprehensive counseling regarding further steps, and the patient was promptly referred to a pediatric endocrinologist for specialized management.

## Introduction

The clitoris, a complex organ whose intricate anatomy has only been fully elucidated recently through advanced imaging methods, can undergo abnormal enlargement known as clitoromegaly. This enlargement, whether congenital or acquired, often arises from an excess of androgens during fetal development, infancy, or adolescence. While obvious cases of clitoromegaly in individuals with ambiguous genitalia are easily identifiable, subtler presentations may go unnoticed. There are numerous reports detailing clitoromegaly, with or without accompanying clinical or biochemical signs of androgen excess [1].

Among individuals with the chromosomal configuration 46,XX, the most common cause of neonatal clitotomegaly is hormonal, specifically the disruption of normal adrenal gland functioning, called congenital adrenal hyperplasia (CAH), contributing to virilization of the external genitalia [2]. 

CAH, most commonly caused by 21-hydroxylase deficiency, is a significant genetic endocrine disorder that requires early recognition due to its potentially life-threatening and life-altering consequences. Its clinical importance lies in the profound effects it can have on the health and development of affected individuals, especially when diagnosis is delayed. [3] 

In its classic form, CAH disrupts the normal production of cortisol and often aldosterone, leading to an overproduction of androgens. In newborns with the salt-wasting form, which represents the most severe subtype, the inability to retain sodium and excrete potassium can result in adrenal crises within the first few weeks of life. These crises, characterized by dehydration, vomiting, hypotension, electrolyte imbalances, and potentially fatal shock, are entirely preventable with timely diagnosis and treatment. However, when CAH goes undiagnosed in the neonatal period, these crises may be mistaken for common viral illnesses or sepsis, delaying life-saving steroid replacement therapy [3].

Another major risk of delayed diagnosis involves newborn girls with significant virilization. These infants may present with ambiguous genitalia and, in the absence of early testing, may be incorrectly assigned male sex at birth. This not only leads to delays in proper hormonal treatment and surgical counseling but can also cause profound psychosocial distress for families. Even in less severe cases, untreated CAH can lead to progressive virilization, rapid bone maturation, short adult stature, menstrual irregularities, infertility, and psychological burden in both sexes [3]. 

Because of these serious risks, many countries have integrated CAH into their national newborn screening programs. These programs, typically using a dried blood spot to measure 17-hydroxyprogesterone levels, enable early diagnosis, often before symptoms develop. In countries such as the United States, Australia, Germany, Sweden, and Japan, routine screening has significantly reduced the incidence of adrenal crises and prevented misdiagnoses related to genital ambiguity. While early screening does carry some limitations, such as false positives in premature infants, second-tier testing methods like steroid profiling by tandem mass spectrometry are enhancing the accuracy of detection [3]. 

The inclusion of CAH in newborn screening reflects a broader international recognition of the condition’s burden and the necessity of prompt intervention. Delayed diagnosis not only endangers physical health but also impacts psychosocial outcomes, long-term fertility, and quality of life. Early identification, by contrast, opens the door to appropriate glucocorticoid and mineralocorticoid therapy, careful sex assignment decisions, and long-term endocrine follow-up that together dramatically improve patient outcomes [3].

## Case presentation

In this presented case report, we describe a newborn patient who was delivered at a prominent obstetrics and gynecology center in Tbilisi, Georgia, exhibiting evident clitoromegaly. 

The patient was born at full term, following an uneventful pregnancy of a healthy 22-year-old mother. Scoring 9/10 on the Apgar scale at one and five minutes, with no apparent anomalies, the newborn weighed 3600 grams and measured approximately 51 cm in length. 

**Figure 1:**
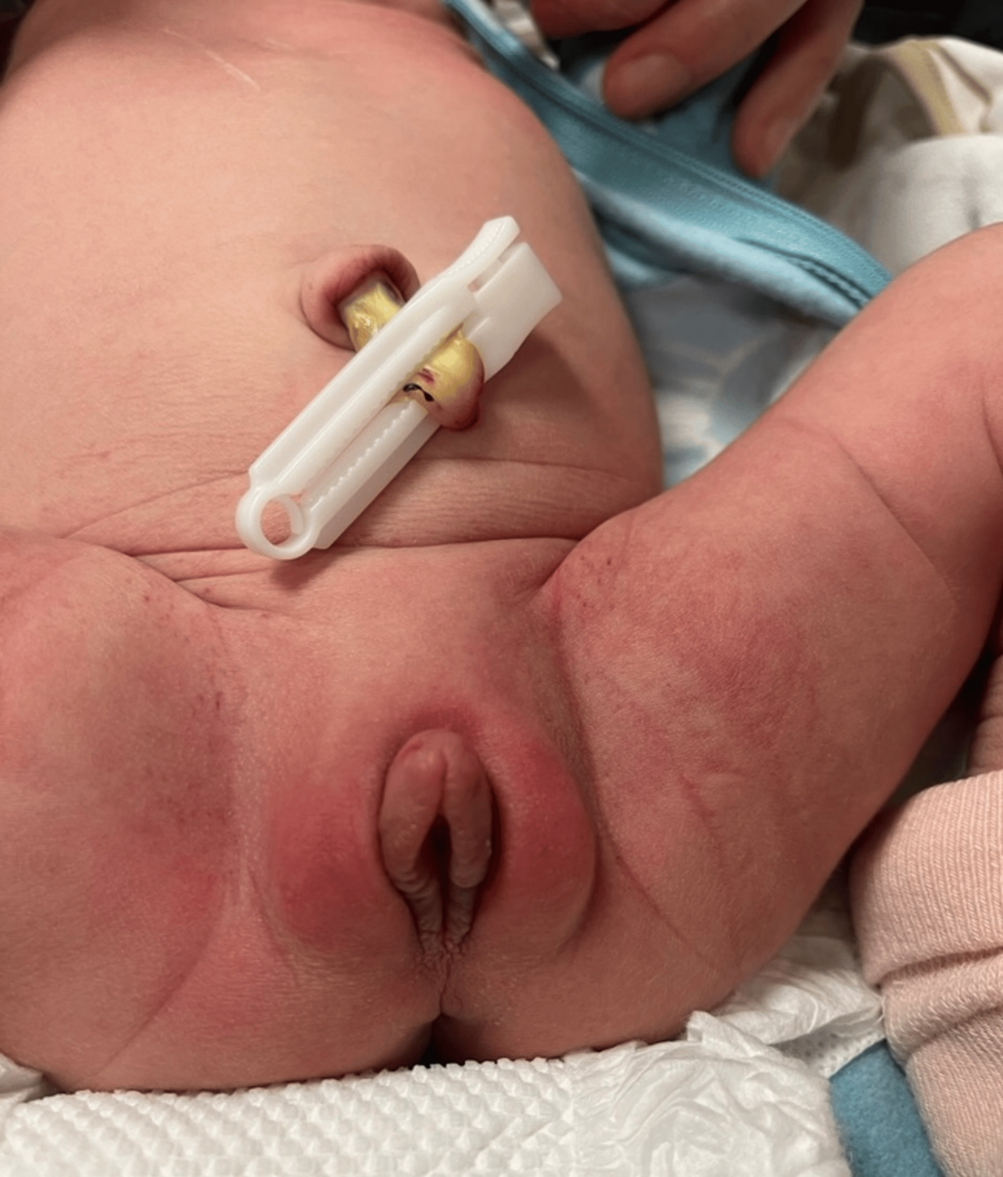
External genitalia appearance 12 hours postpartum Photo taken by the author of the article - Mariam Svanadze

**Figure 2:**
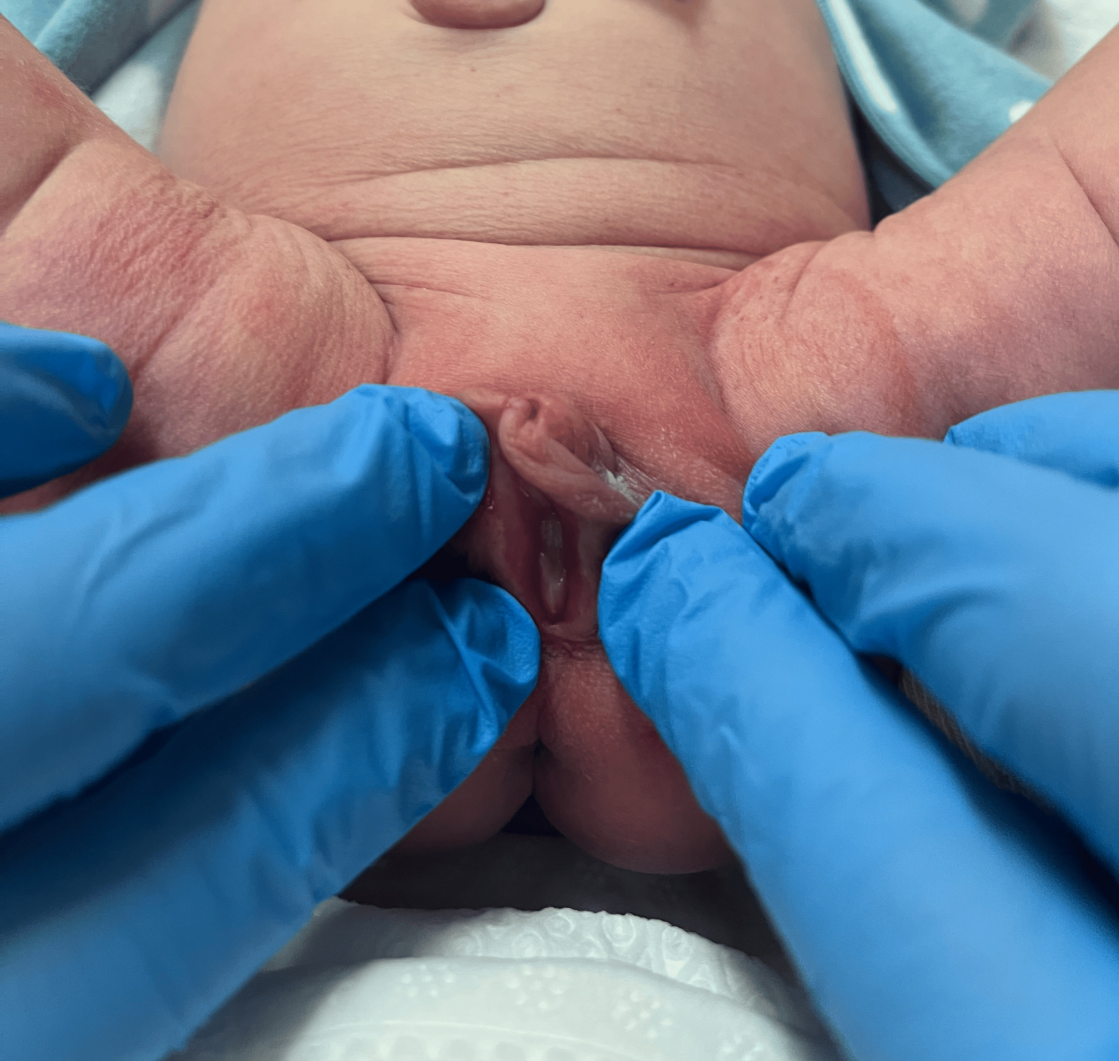
External genitalia appearence 12 hours postpartum Photo taken by the author of the article - Mariam Svanadze

However, the genitalia of the patient exhibited notable abnormalities, characterized by fused, hyperemic, and enlarged clitoris measuring 13 mm in length and 10 mm in width, along with similarly affected labia majora (Fig. 1). Despite the presence of all external female structures and an anteriorly located urethra, these structures were notably hypertrophied and hyperemic (Fig. 2).

At birth, the external genitalia of a newborn provide important clues to the infant’s underlying endocrine health. One of the most carefully examined features in female newborns is the clitoris, as clitoromegaly may be the earliest visible sign of androgen excess [4].

In a healthy term female newborn, the clitoris is typically small, often hidden beneath the clitoral hood. Most newborn girls have a clitoral length of less than 5 mm and a clitoral width of less than 4 mm. These values fall within the normal range and reflect the absence of prenatal androgen exposure. When the clitoris exceeds these dimensions, particularly when the length measures greater than 10 mm, this may indicate virilization, commonly seen in classic 21-hydroxylase deficiency, the most frequent form of CAH [4].

A large, exposed clitoris, especially when accompanied by partial labial fusion, should prompt urgent evaluation, including a karyotype and serum 17-hydroxyprogesterone testing. However, clinicians must also consider ethnic and gestational age variations, as premature infants may have slightly different reference norms. Still, even in preterm infants, marked clitoromegaly warrants endocrine assessment [4].

Throughout history, various methodologies have been employed to assess the dimensions of the clitoris and to ascertain deviations from typical size. Earlier approaches predominantly emphasized measurements of crosswise and lengthwise widths [1]. A contemporary method, proposed by Brodie et al. [5], advocates for assessing the length of the hood alongside crosswise width. The latter method was adopted in the present case. 

**Figure 3:**
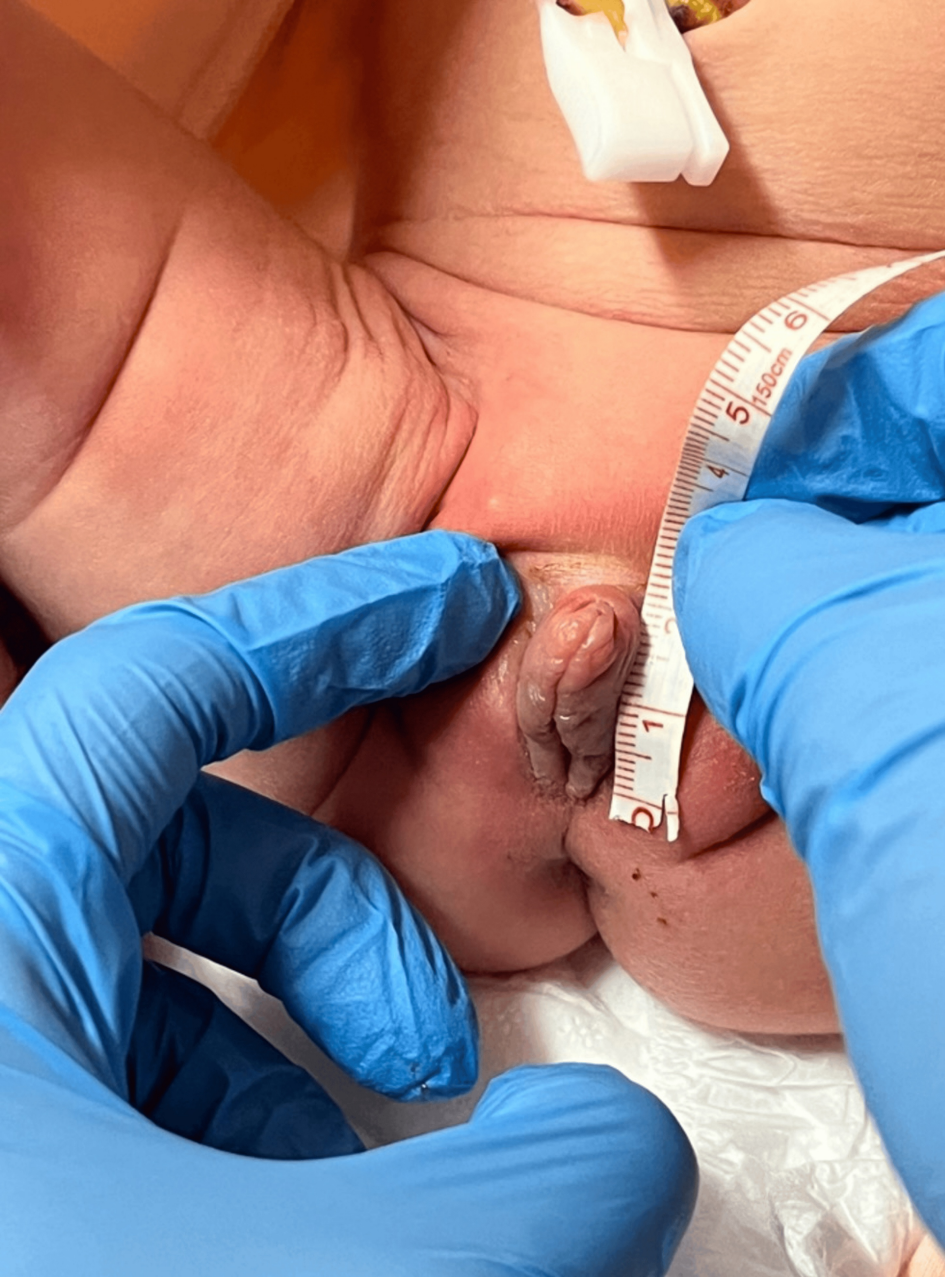
External genitalia on the second day postpartum Photo taken by the author of the article - Mariam Svanadze

**Figure 4:**
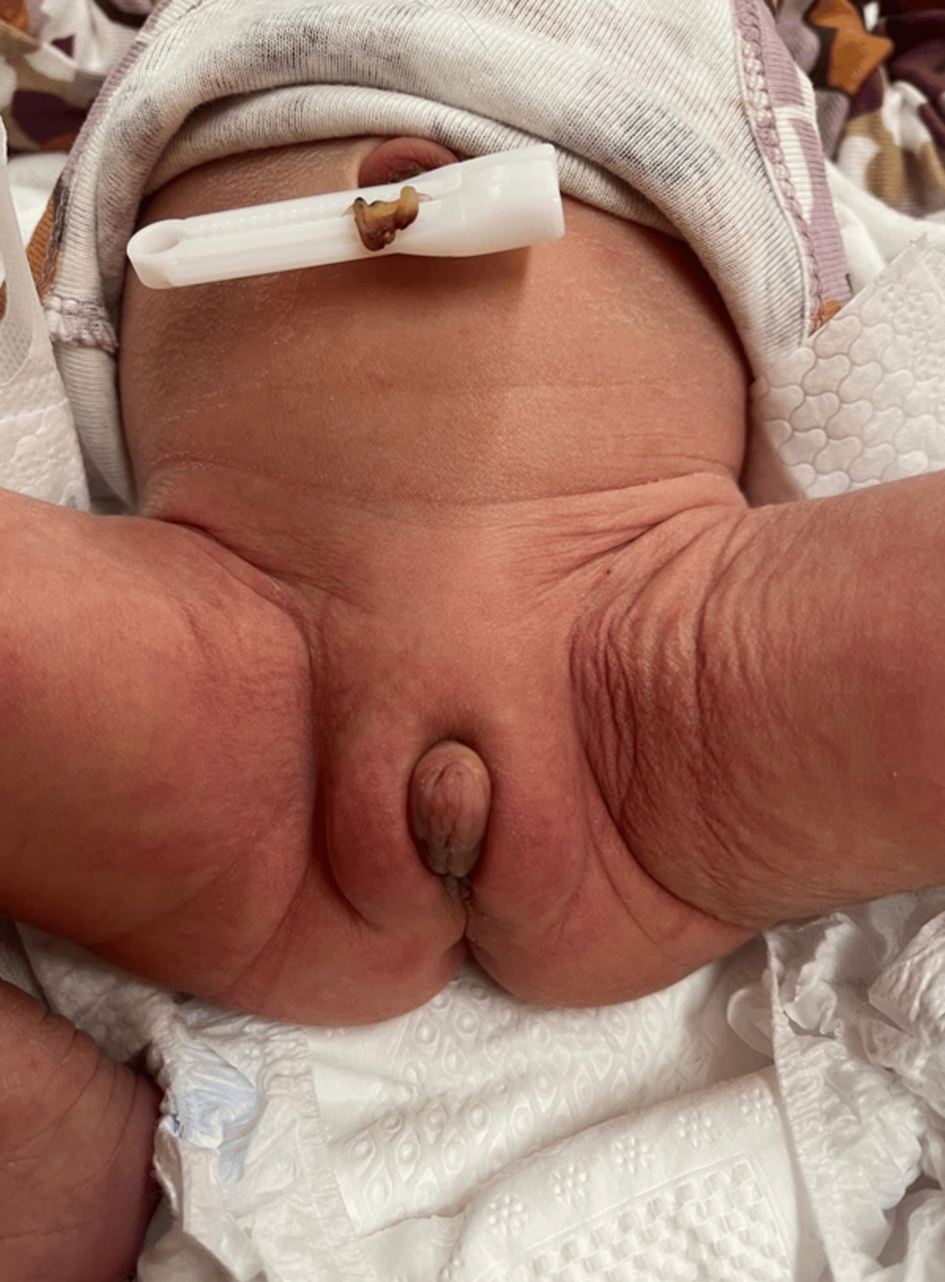
External genitalia on the second day postpartum Photo taken by the author of the article - Mariam Svanadze

The investigative process commenced to ascertain the underlying diagnosis. A thorough review of the medical history revealed no informative clues, with the pregnancy proceeding without complications, no utilization of hormonal medications, and an uncomplicated labor resulting in a full-term birth. An ultrasound performed on the second day postpartum identified a present uterus measuring 40 × 14 × 13 mm (Fig. 3, Fig. 4), while ovaries were not visualized, consistent with expectations for this timeframe. Subsequently, a genetics specialist recommended karyotyping of the patient and consultation with an endocrinologist to confirm or negate the diagnosis of CAH. Meanwhile, clinical findings stayed the same - only the hyperemia of the labium majus decreased. 

Laboratory analyses conducted at 10 days postpartum revealed pertinent findings, notably elevated renin levels and decreased cortisol levels, indicative of disrupted adrenal hormonal pathways and diminished corticosteroidal production.

Specifically, cortisol level was equal to 11 mcg/L, when the normal value for this age is 62-194mcg/L; renin level was 160 ng/L, greatly differing from the normal value of 2.3-133.6 ng/L [6]; and 17-hydroxyprogesterone and electrolyte levels were normal. 

While elevated 17-hydroxyprogesterone (17-OHP) is the key biochemical marker of classic 21-hydroxylase deficiency, levels may be falsely normal if measured within the first 24-48 hours of life. This can be due to immature adrenal steroidogenesis or assay variability. Thus, in clinically suspicious cases, repeat testing and further endocrine evaluation are essential, even if the initial 17-OHP is normal [3].

Furthermore, clinical manifestations of hyperandrogenism, notably evidenced by clitoromegaly, prompted the diagnosis of possible CAH. 

The patient's parents received comprehensive counseling regarding further steps, and the patient was promptly referred to a pediatric endocrinologist for specialized management. 

## Discussion

The case presented highlights the diagnostic complexity and clinical implications of CAH, particularly in neonates with atypical or incomplete biochemical profiles. Our patient, a full-term 46,XX newborn with evident clitoromegaly and fused labial structures, exhibited no overt signs of salt wasting yet demonstrated elevated renin and markedly reduced cortisol levels. These findings, alongside virilized external genitalia, were highly suggestive of classic 21-hydroxylase deficiency CAH, despite a normal 17-hydroxyprogesterone (17-OHP) level.

The degree of clitoromegaly in this case - 13 mm in length and 10 mm in width - substantially exceeded established reference values for term neonates. When the clitoris exceeds 10 mm in length, it is considered highly indicative of prenatal androgen exposure and warrants further investigation for disorders of sex development, particularly CAH [4]. This patient’s measurements, coupled with partial labial fusion, meet criteria for Prader stage III virilization, which is consistent with the classic form of CAH [6].

Although elevated 17-OHP is the biochemical hallmark of classic CAH, early neonatal measurements may yield falsely normal results due to immature adrenal enzymatic activity, delayed hormonal surges, or assay interference. In the present case, 17-OHP measured on day 10 was within normal limits; however, the literature documents several cases of clinically evident CAH where 17-OHP levels are normal or borderline in the early neonatal period. As such, reliance on a single hormonal snapshot can be misleading, and repeat measurements or second-tier assays should be considered [7].

The markedly low cortisol level and elevated renin activity in our patient provide strong diagnostic clues. Cortisol deficiency leads to compensatory ACTH elevation, adrenal hyperplasia, and androgen excess. Renin elevation may precede overt electrolyte abnormalities and is an early marker of mineralocorticoid insufficiency in salt-wasting forms of CAH [1]. The absence of hyponatremia or hyperkalemia at this stage suggests early disease evolution, as similar cases have shown hormonal disturbances emerging days before metabolic decompensation [8].

Importantly, the presence of ambiguous genitalia in a 46,XX neonate should prompt urgent endocrine and genetic work-up. Misassignment of sex at birth has profound consequences, both medical and psychosocial, and delayed diagnosis can lead to irreversible virilization, accelerated skeletal maturation, short stature, and infertility [9]. In countries with universal CAH screening programs, such as the United States and Sweden, early diagnosis has significantly reduced morbidity and sex assignment errors [10]. In Georgia, where such screening is not standard, heightened clinical awareness is critical.

This case underscores the value of a systematic, clinically driven approach in evaluating newborns with ambiguous genitalia, even when initial hormonal assays appear inconclusive.

## Conclusions

This case illustrates the importance of early, systematic evaluation in infants with clitoromegaly, even when some of the initial labs appear normal. Clinical vigilance, repeat testing, and early endocrine referral are critical for timely CAH diagnosis. Multidisciplinary care and clear communication with families enhance outcomes. Expanding newborn screening, provider education, and access to diagnostic resources can reduce delays and improve care for disorders of sex development. 
